# Growing Season Carbon Dioxide Exchange in Flooded Non-Mulching and Non-Flooded Mulching Cotton

**DOI:** 10.1371/journal.pone.0050760

**Published:** 2012-11-30

**Authors:** Zhi-guo Li, Run-hua Zhang, Xiu-jun Wang, Fang Chen, Chang-yan Tian

**Affiliations:** 1 State Key Laboratory of Desert and Oasis Ecology, Xinjiang Institute of Ecology and Geography, Chinese Academy of Sciences, Urumqi, China; 2 Wuhan Botanical Garden, Chinese Academy of Sciences Key Laboratory of Aquatic Botany and Watershed Ecology, Chinese Academy of Sciences, Wuhan, China; 3 Wuhan Vegetable Research Institute, Wuhan, China; DOE Pacific Northwest National Laboratory, United States of America

## Abstract

There is much interest in the role that agricultural practices might play in sequestering carbon to help offset rising atmospheric CO_2_ concentrations. However, limited information exists regarding the potential for increased carbon sequestration of different management strategies. The objective of this study was to quantify and contrast carbon dioxide exchange in traditional non-mulching with flooding irrigation (TF) and plastic film mulching with drip irrigation (PM) cotton (*Gossypium hirsutum* L.) fields in northwest China. Net primary productivity (*NPP*), soil heterotrophic respiration (*R*_h_) and net ecosystem productivity (*NEP*) were measured during the growing seasons in 2009 and 2010. As compared with TF, PM significantly increased the aboveground and belowground biomass and the *NPP* (340 g C m^−2^ season^−1^) of cotton, and decreased the *R*_h_ (89 g C m^−2^ season^−1^) (p<0.05). In a growing season, PM had a higher carbon sequestration in terms of *NEP* of ∼ 429 g C m^−2^ season^−1^ than the TF. These results demonstrate that conversion of this type of land use to mulching practices is an effective way to increase carbon sequestration in the short term in cotton systems of arid areas.

## Introduction

Management and policies to increase the carbon (C) sink of agricultural soils have gained more and more attention, and it can be accepted as one of the greatest potential methods to sequester C in terrestrial ecosystems [1], [2]. Different agronomic practices have been evaluated and recommended to reduce CO_2_ emissions and increase C in soils such as the elimination of tillage, continuous cropping, cover cropping, using legumes in rotation and manure and crop residue application [3]–[5]. For example, Sperow *et al*. [6] suggested that a change from conventional tillage to no-tillage on all currently annually cropped areas (129 Mha) in the United States could result in 47 Tg C sequestered annually. Vleeshouwers and Verhagen [7] identified animal manure incorporation and the use of straw (or crop residues) could sequester as much as 0.19 and 0.15 Mg C ha^−1^ yr^−1^, respectively. Although several studies have advanced the notion that changes in soil C stocks are associated with changes in crop management or in land use [1], there is still a lack of information regarding the potential C sequestration resulting from the conversion from traditional non-mulching cultivation to mulching cultivation [8].

Recently, newer cultivation technologies incorporating mulching with plastic film together with drip irrigation (PM) have been shown to increase soil temperatures and conserve soil moisture [9], [10], resulting in increased crop production. PM cultivation is now widely applied in China [11], [12] with land areas of this type of cultivation reaching 1,000,000 ha in northwestern China in 2009 [13]. It has replaced traditional cultivation (TF) involving flood irrigation with no mulching, which was the main cropping system used in dry land agriculture in northwest China before 1980 [12]. These large land use changes alter the soil microenvironment and have significant effects on the C balance of agro-ecosystems [1]. Understanding the extent to which the conversion of TF to PM contributes to the C cycle is important for developing C management strategies in dry land areas of China.

At present, there are few studies where a complete ecosystem approach (net ecosystem productivity [*NEP*], net primary productivity [*NPP*] and soil heterotrophic respiration [*R_h_*]) is used to compare the C fluxes in traditional and modern cultivations of agro-ecosystems. In this study, a full C balance approach was used to investigate short-term effects (2 years) of cotton (*Gossypium hirsutum* L.) conversion from TF to PM on *NEP*. This study aims to quantify and contrast the amount of C sequestered by the plant biomass and the C lost through soil respiration in PM and TF cotton fields, and to estimate the net gains/losses of C due to the conversion of PM to TF in dry land cotton fields.

## Materials and Methods

### 2.1. Site Description

The study site was located at the Fukang Station of Desert Ecology (87° 56′ E, 44° 17′ N) of the Chinese Academy of Sciences at an elevation of 461 m [14]. The soil type is a clay loam with a bulk density of 1.56 g cm^−3^. Values of pH, electrical conductivity and total organic carbon at 0–10 cm are 8.42, 2.08 ms cm^−1^ and 5.64 mg C g^−1^, respectively. In 2009 and 2010, daily mean air pressures ranged from 922.9 to 996.3 hPa. The daily mean air temperature had a maximum of 32°C, a minimum of −21°C, and a mean of 9.28°C. Daily mean air relative humidity showed large fluctuations, varying from 27.2 to 100%. Total annual precipitation was 164.2 mm in 2009 and 166.2 mm in 2010, slightly higher than the long-term average of 160 mm for the last 30 years.

### 2.2. Field Experiment

The field site has been reclaimed for 15 years from desert, and has been continuously planted cotton under plastic film mulching cultivation. In this field, we designed two treatments during the cotton growing seasons of 2009 and 2010: (1) the traditional cultivation system (TF) of flood irrigation with no mulching; and (2) a modern cultivation system using plastic film mulching with drip irrigation (PM). Each treatment was replicated three times in a randomized complete block design using plots 15×8 m. The cotton seeds (C.V. Xinluzao No. 6) were sown on April 21, 2009, and April 28, 2010. For the PM treatment, a high density and airtight transparent polythene film (0.01–0.02 mm thick, 1.25 m wide) was placed over the soil surface before sowing. The seeder made small holes (0.02×0.02 m) at 0.1 m intervals within a row in the plastic film and seeds were placed into the holes, and then each hole was covered with soil. Four rows were sown on each strip of plastic film. For the TF treatment, the plants were sown as for the PM treatment. For both treatments the planting density was 266 667 plants ha^−1^. Irrigation models were used: drip irrigation under mulching for the PM treatment and flooded irrigation for the TF treatment, and the timing of irrigation and its volume followed the local commercial practice. Nitrogen fertilizer (110 kg N ha^−1^) and phosphorus fertilizer (72 kg P_2_O_5_ ha^−1^) were applied to both treatments, and weeds were controlled by hand.

### 2.3. Microclimate

During the growing season of 2009 and 2010, measurements of air temperature at 30 cm and soil temperature at 10 cm in planted sites and non planted sites with and without chambers, were made for each sample by using a SN2202 digital thermo detector (Sinan Instruments Plant of Beijing Normal University, Beijing). At the same time, soil water content at 10 cm in each plot was measured with the oven drying method (105°C for 48 h).

### 2.4. Growth Analysis

From May 10 to October 20 in 2009 and 2010, five 50×50 cm quadrats were randomly selected from both the PM and the TF every 25 days after planting. The above ground biomass in each quadrat was clipped to ground level and put in large brown paper bags. The below ground biomass was determined by the excavation of 50×50 cm pits at the same time to a depth of 70 cm in 20 cm increments. The live below ground biomass in each quadrat was collected and put in large brown paper bags. Biomass samples were washed in a series of five de-ionized water baths and weighed after oven drying for 5 days at 75°C. Grain yield, for each plot, was determined on three 4-m^2^ subplots. Organic C was measured by oxidation with K_2_CrO_7_ according to the method of Liu *et al*. [15].

### 2.5. Net Primary Productivity

In this study, the cotton *NPP* was calculated by the measurement of above and below ground biomass during the different cotton periods and the C content using the following equation:
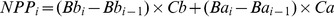
(1)where *i* is the measurement time of the plant biomass, *Bb* is the below ground biomass (g m^−2^), *Cb* (g C g^−1^) is the carbon content of the below ground biomass, *Ba* is the above ground biomass (g m^−2^), *Ca* (g C g^−1^) is the carbon content of the above ground biomass.

### 2.6. Soil Heterotrophic Respiration (R_h_) Measurements

Soil heterotrophic respiration was measured at midday using a closed opaque chamber (0.5×0.5×0.5 m), which has been a method used in previous studies [16], [17]. The chamber was equipped with a small circulation fan and a gas channel, which was a PVC tube with a three-way stopcock. A stainless steel frame with a water groove (0.5×0.5×0.05 m) was inserted into the soils, and the chamber was put into a groove that was filled with water before each gas sampling to ensure an airtight seal.

Three steel frames were placed between cotton rows in each replicate. Nylon nets (200 cm long×50 cm height, mesh size 0.25 mm) were buried around the frame to prevent roots entering the soil below the frames. When conducting gas sampling, the gas was collected mostly between 9 and 11 am every other week. The fluxes measured during this time in the morning are regarded to be basically representative for the daily average fluxes [18], [19]. About 70 mL of gas was aspirated from the chamber at 30-s intervals for 150 s after capping. Gas samples were collected in polyethylene coated aluminum gas bags and taken into the laboratory soon after sampling. CO_2_ concentrations were analyzed using a modified gas chromatograph (Agilent 4890D) equipped with an electron capture detector and a hydrogen flame ionization detector. The flux of soil heterotrophic respiration was calculated as follows:
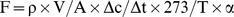
(2)where *F* (g C m^−2^ h^−1^) refers to flux of soil heterotrophic respiration, *ρ* is the density of CO_2_ (1.98×10^3^ g m^−3^) under standard conditions, *V* (m^3^) and *A* (m^2^) are the volume and bottom area of the chamber, Δ*c* (m^3^ m^−3^) is the change in CO_2_ concentration in the chamber during the period Δ*t* (h), *T* is the absolute temperature and *α* is the conversion factor for CO_2_ to C (12/44).

### 2.7. Growing Season Net Ecosystem Productivity (NEP) Measurements

*NEP* is a comprehensive measure of net carbon accumulation by ecosystems. Growing season *NEP* can be calculated as the difference between the season *NPP* and season *R*_h_:
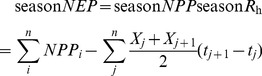
(3)where season *R_h_* represent cumulative soil CO_2_ emissions throughout the cotton growing season; *i* and j are the measurement times of the plant biomass and soil respiration*; X_j_* (g C m^−2^ d^−1^) is the first week CO_2_ measurement and *X_j+1_* (g C m^−2^ d^−1^) is the following week CO_2_ measurement at times *t_j_* and *t_j+1_*, respectively; and n is the final week of CO_2_ measurement during the experiment periods.

### 2.8 Statistical Analysis

Data comparisons between PM and TF sites were performed by one-way ANOVA using SPSS 11.0 software. Averages from all treatments were compared using Fisher’s least significant difference at a probability level of 0.05.

## Results

### 3.1. Soil Temperature and Soil Water Content

**Figure 1:**
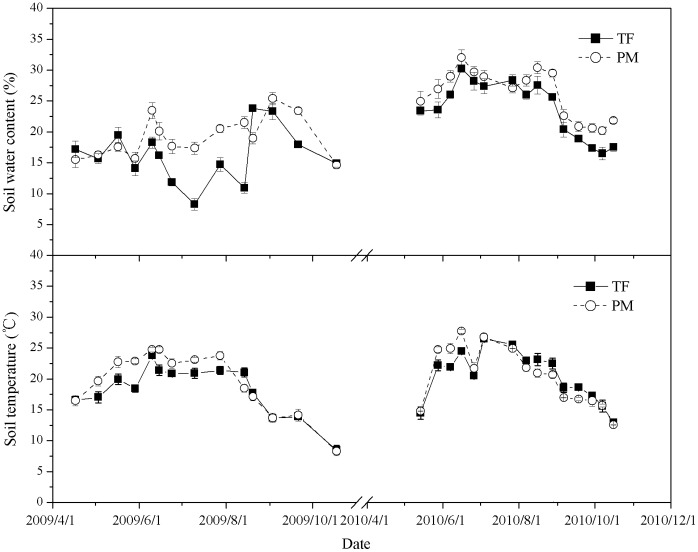
Soil temperature and soil water content measured at 10 cm depth in the traditional cultivation system of flood irrigation with no mulching (TF) and the modern cultivation system using plastic film mulching with drip irrigation (PM) in the cotton growing season of 2009 and 2010.

The mean daily soil temperature showed a marked seasonal trend during the growing season for all years and all sites, and both PM and TF had higher mean annual values in 2010 than in 2009 (Fig. 1). Soil temperature in the PM was significantly higher than in TF during the early growing season (April to July) (p<0.05); however, at the mid-late growth stage (after August), the daily average temperature in PM was obviously lower than that in TF, particularly in 2010. Soil water contents for PM and TF were also higher in 2010 than in 2009 during the growing period. The PM had a significant higher soil water content during the growing period than the TC treatment (p<0.05), but the difference was larger in 2009 than 2010.

### 3.2. NPP

**Figure 2:**
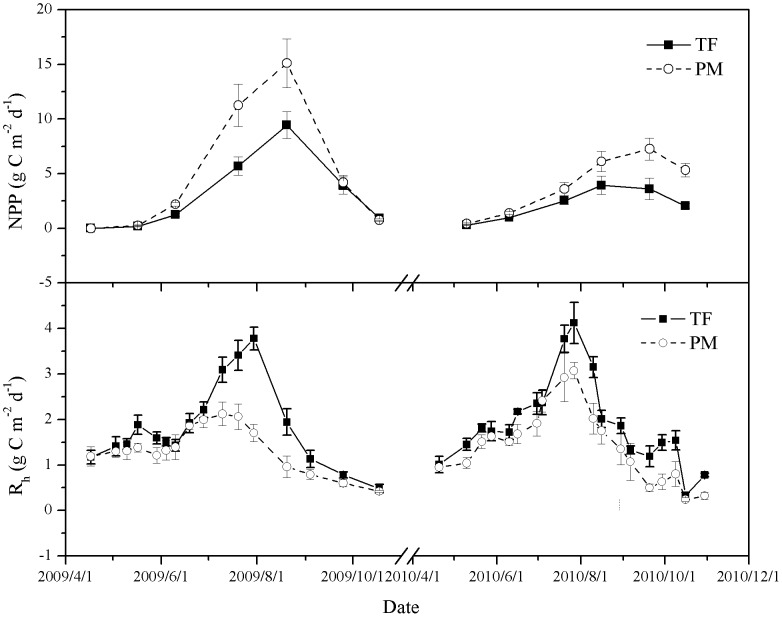
Daily net primary productivity (*NPP*) and heterotrophic respiration (*R*_h_) measured using a closed chamber at the PM site and the TF site in the cotton growing season of 2009 and 2010 (mean±SE).

Daily *NPP* values relative to the whole growing period (April to November) were computed for both land uses. The time series graphs of *NPP* from PM and TF (Fig. 2) showed large fluctuations ranging from 0.17 to 15.12 g C m^−2^ d^−1^ during the whole growing period. *NPP* increased to the highest levels in July and August but remained at a low level during the early (May–July) and late (October) growing season. Overall, the average daily rates of *NPP* in the PM were significantly higher than in the TF, especially during the main growth periods where PM (15.12 g C m^−2^ d^−1^) was approximately 1.5 times higher than TF (9.4 g C m^−2^ d^−1^) (p<0.05). Total *NPP* for the PM during the whole growing season was 1030 g C m^−2^ season^−1^ which was significantly higher than the 689 g C m^−2^ season^−1^ for the TF (Table 1) (p<0.001). In the 2nd year of measurement (2010), total *NPP* on both the PM and PF sites was less than the 1st year (2009) probably because an insect pest infestation broke out during the main cotton growing period (July). However, PM did show a higher total *NPP* than TF, although the difference was smaller.

**Table 1:** Crop management details for the two cultivation systems in the cotton growing season of 2009 and 2010 (±standard deviation where computed, n = 3).

Treatment^a^	Year	Tillage date	Planting date	Applied N (kg N ha^−1^)	Harvest date	Cotton yield (Mg ha^−1^)
PM	2009	Dec.20, 2008	Apr. 21, 2009	110	Oct. 18	4.41±0.37
	2010	Dec. 15, 2009	Apr. 29, 2009	110	Oct. 20	3.89±0.58
TF	2009	Dec.20, 2008	Apr. 21, 2009	110	Oct. 18	3.34±0.64
	2010	Dec. 15, 2009	Apr. 29, 2009	110	Oct. 20	2.85±0.23

a PM = plastic film mulching with drip irrigation; TF = non-mulching with flooding irrigation.

### 3.3. Soil Respiration

Soil respiration also showed strong seasonal variations with occasionally high fluxes of 2.12–4.12 g C m^−2^ d^−1^ between July and August (Fig. 2). The average daily rate of *R*_h_ was 1.86 g C m^−2^ d^−1^ in the PM, significantly higher than the values of 1.39 g C m^−2^ d^−1^ in the TF (p<0.05). During the two growing seasons, the total soil CO_2_ flux in the PM was lower than that in the TF (485 and 663 g C m^−2^, respectively). Thus, the conversion from TF to PM caused a decrease in soil C emissions of approximately 89 g C m^−2^ in one growing season.

### 3.4. Growing Season NEP

Between years, the values of *NEP* from PM and TF varied largely owing to the large fluctuation of *NPP* for both sites. Comparing *NEP* from the two sites, PM was 479 and 379 g C m^−2^ season^−1^ higher than TF in 2009 and 2010 growing season, respectively. Over the two growing periods, PM had a net C uptake of 1234 g C m^−2^ and TF had a net C uptake of 376 g C m^−2^ from the atmosphere (Table 2).

**Table 2:** Carbon balance components for the PM and TF fields in the cotton growing season of 2009 and 2010 (±SD, n = 3).

Date	Site	*NPP* a g C m^−2^	*R_h_* b g C m^−2^	*NEP* c g C m^−2^
2009.4–2009.11	PM	1030±155 a	214±33 b	816±49 a
	TF	648±96 b	311±29 a	337±21 b
2010.4–2010.11	PM	689±97 a	271±19 b	418±15 a
	TF	391±73 b	352±32 a	39±19 b
Total	PM	1719±121 a	485±31 b	1234±72 a
	TF	1039±78 b	663±49 a	376±49 b

a *NPP* = net primary productivity;

b *R_h_* = heterotrophic respiration;

c *NEP* = net ecosystem productivity; In a row, values with the different letters are significant different at 5% level based on Fisher’s least significant difference tests.

## Discussion

In cropland, improved practice can reduce net CO_2_ emissions from soils and stabilize and/or increase C sequestration in the soil, and is being accepted as the best potential method to assist in meeting the demands of an international C credit system [1]–[4]. In this study, the consequences of modern and traditional cultivation management options for C fluxes were investigated using a plastic film mulching management with drip irrigation, versus a non-mulching management with flood-irrigation. It was assumed that all differences between the mulching and non-mulching cropland plots resulted from the contrast in management.

The *NPP* (g C dry matter produced pre unit leaf area and time), referred to as the net CO_2_ uptake by canopy, is a primary determinant of the C flux into the terrestrial biosphere [20]. PM significantly increased cotton *NPP* compared with TF, although the difference between PM and TF was smaller in the 2nd year owing to an insect pest infestation. This PM effect was also found in the earlier results for cotton in the Yellow River Delta China [21], and other crops such as wheat in New Delhi, India [22], and tomato in Mancha, Spain [23]. On the PM site, suitable soil conditions could be established with rational fertilizer use [24], high soil temperature and soil moisture [10], and better weed control [25], which could increase the photosynthetic capacity of crop leaves and lead to an increase in plant C content. Li *et al*. [11] and Chakraboyty *et al*. [22] attributed the higher *NPP* in the PM to the improvement of water use efficiency compared with TF. The increase of water use efficiency in PM is reflected in two aspects: prolonging the soil water retention time in soil by reducing soil water evaporation, and exploiting deep soil water. These changes reduced the water stress, triggered deep rooting growth and improved plant photosynthesis, which resulted in the obvious increase in *NPP* at the PM sites. Liu *et al*. [26] and Li *et al*. [11] also reported that the higher soil temperature of PM, particular at early seedling growth, reduced cell damage from early season chilling and produced larger and more vigorous seedlings compared with TF, and these seedlings then sequestered more CO_2_ from the atmosphere during the growth period.

Besides the obvious increase in *NPP* (C input), PM also changed soil CO_2_ fluxes (C output). Conversion from PM to TF greatly altered soil environment factors such as temperature, plant and microorganism activity, and soil organic carbon content. These factors regulate soil CO_2_ production through biochemical action, autotrophic and heterotrophic respiration [27], [28], and other soil properties such as soil water content and soil porosity, which affects the transfer of CO_2_ in the soil profile [29]. In this study, the soil surface CO_2_ flux was significantly lower in PM than in TF during the whole growth period. The result was opposite to what was expected, because previous studies reported that higher soil temperature and soil water content due to mulching increased soil respiration. The lower CO_2_ in PM may be related to the soil properties. The soils in the present study sites were rich in calcium ions and had a high pH of 8.42 [30], together with a higher soil water content, which possibly led to a favorable soil environment for the formation of carbonate or organo-mineral complexes [31]. Moreover, mulching decreases wind disturbance, which can induce pressure pumping and vertical advection and increase rates of CO_2_ exchange between soil and the atmosphere over and above rates permitted by molecular diffusion alone [32].

*NEP*, an index of ecosystem function related to CO_2_ uptake depends on two contrasting processes: *NPP* and *R_h_*. Due to lower *R_h_* than *NPP* in arid cropland, the mean *NEP* values of 188–617 g C m^−2^ season^−1^ in these cotton systems were both positive and much greater than those observed at forest sites (Borden forest, Ontario: 65–140 g C m^−2^ [33]; European forest: 175 g C m^−2^ [34]; Harvard forest, MA: 174 g C m^−2^ [35]), and grassland sites ([northern temperate grassland in Alberta, Canada: −18 to 20 g C m^−2^ [36]; Mediterranean annual grassland in California: −30 to 130 g C m^−2^ [37]). As, we reported the *NEP* values with regard to single cotton growing season in this study, not surprisingly, the *NEP* values were higher than those reported on yearly basis in other studies. The growing season *NEP* values on the irrigated and rainfed maize sites at the University of Nebraska Agricultural Research and Development Center range from 600–800 g C m^−2^, which approach those of cotton in our study [38]. In addition, in this region, there is plenty of sunshine during growing season, and hence we used the high density cultivation mode. This mode could produce a higher level of *NPP.* These reasons eventually led to higher *NEP* than in other studies.

However, as a consequence of an increase in *NPP* and a relative reduction in *R_h_* rates, PM showed a higher *NEP* (C sequestration potential) and sequestered an average of 429 g C m^−2^ season^−1^ more than TF. Similarly, Saroa *et al*. [39], Jia *et al*. [40] and Liu *et al*. [41] reported that net C content in soil significantly increased in soils following the initial conversion to PM. However, these results are in contrast with Zhang *et al*. [42], who measured significant net losses of SOC in PM soil due to accelerated decomposition of organic matter under the prevailing conditions of higher soil temperature and moisture. The different C content results after conversion to PM were probably due to differences in soil properties and crop species. In the present study, crop soils are rich in calcium ions, have a high pH (8.42) and have a lower SOC content [30], which provided conditions for the formation of carbonate or organo-mineral complexes [31]. Also, cotton has a larger biomass and deeper root systems that was more suitable for sequestering larger amounts of C into the soil profile [43], [44].

Despite these analyses, mulching cultivation still has a relatively finite C sequestration potential. According to results reported by Li *et al*. [45] and Chen *et al*. [46], mulching increased the amount of CO_2_ released from soil because the higher soil temperature accelerated the decomposition of soil organic matter. To improve the performance of C sequestration by the PM ecosystem, we suggest that the mulching cultivation system should be integrated with other highly advanced technological methods such as using no-till practices [47], planting biofuel crops with a greater biomass [48], adopting practices that encourage deep rooting of crops that allocate more C and nutrients deep into the soil profile [49] or adding black biochar into the soil to fix more CO_2_ from soil respiration.

### Conclusion

This study shows that conversion from mulching cultivation to non mulching cultivation in the short term increased the share of cotton *NPP* (C input) and decreased the soil surface CO_2_ flux (C output) by ∼30% for the whole growing season. As a consequence, there was a net increase of C in terms of *NEP* of ∼429 g C m^−2^ season^−1^ after converting to PM cultivation. Nevertheless, to make the mulching cultivation sequestrate more C from the atmosphere into the soil, it was suggested that other C fixing practices are used in conjunction with the mulching cultivation. Furthermore, in order to assess the overall greenhouse gases balance after the conversion from PM to TF, experimental measurements on N_2_O and CH_4_ fluxes are needed.
